# Estimated divergence times of *Hirsutella* (asexual morphs) in *Ophiocordyceps* provides insight into evolution of phialide structure

**DOI:** 10.1186/s12862-018-1223-0

**Published:** 2018-07-13

**Authors:** Jiaojiao Qu, Yeming Zhou, Jianping Yu, Jian Zhang, Yanfeng Han, Xiao Zou

**Affiliations:** 1 Key Laboratory of Green Pesticide and Agricultural Bioengineering, Ministry of Education, Guizhou University, Guiyang, 550025 China; 2 Institute of Entomology, Guizhou University, Guiyang, 550025 China; 3 Institute of Fungus Resources, College of Life Sciences, Guizhou University, Guiyang, 550025 China

**Keywords:** *Hirsutella* (asexual morphs), New species, Origin and evolution, Phialide

## Abstract

**Background:**

*Hirsutella* Pat genus, the asexual morphs of the *Ophiocordyceps* Sung, is globally distributed entomopathogenic fungi, which infect a variety of arthropods, mites and nematodes. The fungal species also have shown potential application in the field of biological control, bio-medicine and food development. Although these fungi are synonymized under *Ophiocordyceps*, formal taxonomic assignments remain necessary for classification of species in *Hirsutella*. However, due to the heterogeneity and complexity of *Hirsutella* genus, more detailed taxonomic and phylogenetic analyses are required to address the following subjects: (1) the relationships between the phialide morphological characteristics and phylogenetic information of *Hirsutella* with asexual morphs, (2) the origin and evolution of the phialide structure, and (3) host specificity and fungal pathogenicity.

**Results:**

Five typical phialide structures are summarized, in which the variation in phialide characteristics overlaps well with phylogenetic information. A new member of the special twisted neck clade in the *Hirsutella*-like group, *Ophiocordyceps retorta*, was reported based on these analyses. The molecular clock calibration analysis based on one fossil record revealed that *Hirsutella* (asexual morph) species originated from a common ancestor approximately 102 million years ago (Mya) (Early Cretaceous, Lower Albian) and then resolved into two major lineages. One lineage was typically phialidic, which was a larger shape, including *H. guyana*, *H. nodulosa* and *H. sinensis* clades (86.9 Mya, 95% highest posterior density (HPD): 69.1–101.4 Mya). Another main lineage of the phialides was more diversified and smaller than the former, which included *H. citriformis* and *H. thompsonii* clades (71.9 Mya, 95% HPD: 41.8–99.6 Mya).

**Conclusions:**

Our results showed that certain phialide characteristics of *Hirsutella* were phylogenetically informative for two groups of taxa. The differentiation of the phialides structures in the major clades demonstrated a clear evolutionary path of *Hirsutella* (asexual morph) species, which exhibited two trends depending on the host size. Fungi in one of the groups displayed elongated conidiogenous cells with increased complexity of auxiliary structures from the mycelia. The species in another group reduced the volume of phialides and spores, which might be due to an energy-efficient strategy. These results suggested that a common origin allowed for diversification of given clades into separate niches. The distinct parallel evolutionary path combined with the specific phialides structure might result in the host specificity of *Hirsutella* (asexual morphs). A direct relationship between *Hirsutella* (asexual morphs) and the Cretaceous–Tertiary extinction was not found, which suggested that the diversity of phialides is more likely to be caused by long-term environmental adaptation and evolution rather than dramatic extinction events. This evolutionary result might correspond to the background of important biological and geological events in the late Cretaceous occurring near the divergence times of *Hirsutella* (asexual morphs).

**Electronic supplementary material:**

The online version of this article (10.1186/s12862-018-1223-0) contains supplementary material, which is available to authorized users.

## Background

The genus *Hirsutella* Pat., established in 1892 by Patouillard with *H. entomophila* as the type, includes the asexual morphs of specialized parasites belonging to the family Ophiocordycipitaceae [1, 2]. It is an important fungal genus due to various features, including its potential for use in the biological control of invertebrate pests, nematodes and mites [3, 4]. The fungal species are also a popular traditional medicine and nutritious food in many Asian countries due to the production of several bioactive secondary metabolites [5–9].

To conform the International Code of Botanical Nomenclature (Article 59), many fungal taxa typified by asexual morphs has been proposed suppression of generic names [2, 7, 10, 11]. Currently, as the oldest asexual generic name associated with the ‘*Ophiocordyceps sphecocephala* clade’, *Hirsutella* was synonymized under *Ophiocordyceps*, most of which sporulate on cadaver of the host adult insects [12–14]. However, the genus *Hirsutella* was suggested to be separately- treated due to the complex interrelationship in their anamorphs, which has been applied to a heterogenous group of fungi across several families of Hypocreales [15]. Therefore, additional sampling, more detailed taxonomic and phylogenetic analyses of these species are required to confirm their placement in *Ophiocordyceps* instead of suppression of the generic name continuous due to a lack of knowledge or importance of these fungi. These efforts maybe promote the impending taxonomic revisions of the family Ophiocordycipitaceae [11, 14, 16].

The main taxonomic features of *Hirsutella* (asexual morphs) that distinguish from other asexually typified genera, are the basally inflated phialide in a discontinuous hymenial layer and the conidia embedded in mucous sheaths [17]. Since *Hirsutella* was first described, the morphological features of this genus have expanded to including the possibility of polyphialides lacking significant basal inflation and conidia lacking a mucous coat [11, 15]. According to Hodge [18], the morphological features of *Hirsutella* species are insufficient to determine the evolutionary relationships between species. Therefore, further works on the phylogeny of *Hirsutella* species have been conducted [19, 20]. Simmons et al. [11] attempted to define certain clades and correlate them with morphology. They recognized the genus as five distinct groups: *H. citriformis*, *H. guyana*, *H. nodulosa*, *H. sinensis* and *H. thompsonii*, and tried to lay the ground work for new nomenclature by identifying characters and supported clades for future partitioning of *Ophiocordyceps*. However, no robust pattern was found that based on the host, nor did Hodge [18].

In addition to the trends in host taxa, one morphological character, the structure of phialides, nearly overlaps with phylogenetic information from the phylogenetic tree established in our previous work [21]. Phialides of *Hirsutella* species vary in size, shape and branching. In terms of size, the phialides could be divided into two groups. The phialide in one of the groups is shorter than 25 μm, such as *H. citriformis* [22] and *H. thompsonii* [23], and is long, with an average length of more than 40 μm (even reaching 100 μm in some species) in another group, such as *H. aphidis*, *H. guyana*, *H. illustris* and *H. sinensis*. As for the shape, *Hirsutella* species also display much diversity in phialides. *H. sinensis* has a typical bottle shape with a cylindrical base [24]. *H. nodulosa* exhibits helical twisting at the apex of phialides and warts on phialides and hyphae [25]. *H. citriformis* is mainly represented by a dumpy ovoid base and a single slender neck [26]. *H. thompsonii*, the most widely studied *Hirsutella* species and an important biocontrol agent for mite pests in agriculture, has a cylindrical or round phialide that is small in shape. If the morphological characteristics of these phialides could be consistently associated with the molecular phylogeny, the data would be beneficial and provide evidence for support of revisions to the phylogeny and taxonomic transitions which move forward under the new rules.

As the host-specific entomopathogenic fungi, *Hirsutella* (asexual morphs) species are much different from *Beauveria* and *Metarhizium*, which have more than 700 hosts due to morphological structure and physiological characteristics [20]. Despite the characteristics of single sporulation, low sporulation, slow growth and a unique host insect, *Hirsutella* have been preserved and evolved in the natural environment for a long time, which cause epidemics in their host population, and show remarkably environmental adaptability. It is demonstrated that conidia mucilage, like natural film coatings, contribute to spore surface hydrophobicity and play a positive role in conidial viability of *Hirsutella satumaensis* under adverse environments [27]. The appearance of conidiogenous cells (phialides), such as the position, shape, length and size, are the main identifying characteristics in taxonomy and have been extensively studied. However, it is little understand whether there is a connection between the diversity of phialides and ecological adaptation in host specificity and entomopathogenicity. In addition, since it is difficult to amplify some universal gene fragments for the species, and only partial sequences are available for genetic analysis, few study has been performed to comprehensively analyse the evolution of *Hirsutella* (asexual morphs) at the molecular level [15, 28, 29]. To address these problems, more efforts should be made to supplement more available taxonomic information, to perform accurate phylogenetic research, to estimation the evolutionary timescales using the molecular clock, and to improve understanding of evolutionary processes across all taxonomic levels.

To better understand the internal phylogeny of the *Hirsutella* (asexual morphs) species and the relationship with phialide structure, we conducted a phylogenetic analysis with three loci from 40 species representing a broad diversity of conidiogenous cell structures in this study. On the basis of phylogenetic analysis, one fossil record of *Paleoophiocordyceps coccophagus* G. H. Sung, Poinar & Spatafora [29], which is phylogenetically close to *Hymenostilbe* and *Hirsutella*, was used as a calibration point for a molecular clock analysis to estimate the divergence times of the different lineages of the *Hirsutella* (asexual morphs) species, with particular regard to the phialide structure. We also attempt to reveal the origin, evolution history and relationship with pathogenicity and host specificity of the fungi. In addition, a new member of the twisted phialide neck group in *Hirsutella* (asexual morphs) was added from an epidemiological study on invertebrate insects in China.

## Methods

### Specimens

The new *Hirsutella* (asexual morphs) species was isolated from cadaver of Cochlidiidae larvae (Lepidoptera) collected from Leigongshan Nature Reserve, Guizhou province, China (26° 15’ N, 108° 09′ E), on 12 August 2010 by X. Zou. The specimen and an isolated strain of asexual stage were deposited as GZUIFR-lgs2 and GZUIFR-hir100812, respectively.

### Fungal isolation and culture

The isolated strain GZUIFR-hir100812 was determined with sterile culture as described previously [30, 31]. Briefly, the surface of specimen was rinsed with sterile water, followed by surface sterilization with 75% ethanol. A piece of mycelia from the haemocoel was used to inoculate on potato dextrose agar (PDA) plate that was allowed to grow for 14 days at 16 °C under 12-h light/12-h dark conditions.

### OM and SEM observations

For the asexual conidiogenous structure and conidia observations, fresh hyphae were observed and imaged using an optical microscope (OM, BK5000, OPTEC, USA) after staining with lactic acid phenol cotton blue solution. The captured images were edited and digitally contrasted with Paint Shop Pro v. 5.0.1 (Corel, Ottawa, Canada) [30].

Scanning electron microscopy (SEM) was conducted according to Qu et al. [27, 30]. Briefly, the fresh fungal hyphae were collected from PDA cultures and fixed with 4% glutaraldehyde at 4 °C overnight. Then the sample was washed three times with phosphate buffer solution (PBS) for 10 min/time. The fixed hyphae and conidia were dehydrated using 50, 70, 90 and 100% alcohol for 10 min/each level, and with supercritical carbon dioxide. After sprayed with gold, the samples were examined via SEM (S-3400 N, HITACHI, Japan) and imaged.

### DNA extraction, PCR amplification and sequencing

Mycelia (0.05–0.1 g) were harvested from PDA plates and used for genomic DNA extraction with a Fungi DNA isolation Kit according to the manufacturer’s instructions (OMEGA Bio-Tek, USA). Sequences from two nuclear loci, the large subunits of the rDNA (LSU) and the internal transcribed spacers (ITS1–5.8S rDNA–ITS2 region, ITS), and the transcription elongation factor-1α (TEF), were used to phylogenetic analyses. The ITS ribosomal and LSU region were amplified using PCR with primers ITS1/ITS4 and LRor/LR5 respectively [11, 32–34]. The TEF gene was amplified with the primers EF1T and 1567R [35]. PCR product was sequenced and new sequences were submitted in GenBank as shown in Additional file 1: Table S1 (the GenBank access numbers in bold).

### Molecular phylogeny

According to our previous phylogenetic studies, the representative taxa of *Hirsutella* members from the major clades were chosen to construct a phylogeny [21, 32]. A total of 40 taxa were selected to represent the morphological and ecological diversity of *Hirsutella* (asexual morphs), including the outgroup taxa *Purpureocillium* (*Cordyceps cylindrica*) and *Drechmeria* (*Drechmeria gunnii*), which are also classified into Ophiocordycipitaceae [7]. The GenBank accession numbers and strain voucher information are shown in Additional file 1: Table S1. DNA sequences were aligned with the Clustal X ver. 2.0 software as described previously [30]. A combined dataset with the three regions, consisting of LSU (572 bp), TEF (581 bp) and ITS (577 bp) were analysed. The Akaike information criterion (AIC) in jModelTest 2.1.7 was used to select nucleotide substitution models for each three partition [36, 37]. Maximum likelihood (ML) phylogenetic analyses of the combined dataset with partition model parameters in RAxML ver. 8.0 was conducted to determine the best tree topology and bootstrap support values from 500 search replicates [38], which are summarized in FigTree v 1.4.2. Bayesian posterior probabilities (BPPs) were estimated with the same partition parameters in an analysis conducted in MrBayes v 3.1.2 [39], in which two runs of four chains each were simultaneously executed for 5,000,000 generations, with sampling every 500 generations [30]. TreeGraph ver. 2 was used to compute BPP from a summary of 7500 trees retained after a burn-in of the first 2500 trees collected.

### Divergence time estimation of phialide structures in *Hirsutella* (asexual morphs)

A tree was generated with the aligned sequence data using the MCMC (Markov chain Monte Carlo) process to infer divergence times with BEAST ver. 2.2.1 [40]. One internal calibration point corresponding to the fossil record, *P. coccophagus*, a fungal parasite of a scale insect from the Early Cretaceous (Upper Albian) (99–105 Mya), was used in the analysis as soft constraints following a uniform limitation [29, 41]. The root node, which represented the rise of *Ophiocordyceps*, was set at 99–105 Mya [29]. In order to estimate nucleotide substitutions, which were used to count branch lengths and variance-covariance structure of the branch length with the estbranches program, the baseml program of PAML ver. 3.14 with GTR + G was applied to independently calculate each gene as recommended [29, 42].

The gamma-distributed rate variation and a proportion of invariant sites of heterogeneity model were applied with base frequencies estimated during the analysis [43]. A relaxed molecular clock using the uncorrelated log-normal model (mean of root age: 102 with a standard deviation (SD) of 1.5, 0.001 for root rate with an SD of 0.0011; 0.005 for rate autocorrelation with an SD of 0.005) was applied with a Yule process speciation prior for branching rates [29]. The Metropolis-coupled Markov Chain Monte Carlo (MCMC) was run for 10 million generations following a burn-in of 1 million generations and was sampled every 1000 generations. Three replicate runs were performed to check the convergence of the MCMC. The measures of effective sample sizes (ESS) were used to determine the Bayesian statistical significance of each parameter (ESS > 200). Posterior ages were nearly identical among the three analyses, which was consistent with the convergence of the Bayesian dating analyses. Mean parameter estimates and 95% highest posterior densities were determined by analyzing the combined BEAST tree files using Tracer v.1.7 [44].

## Results

### Phylogenetic analysis

**Fig. 1:**
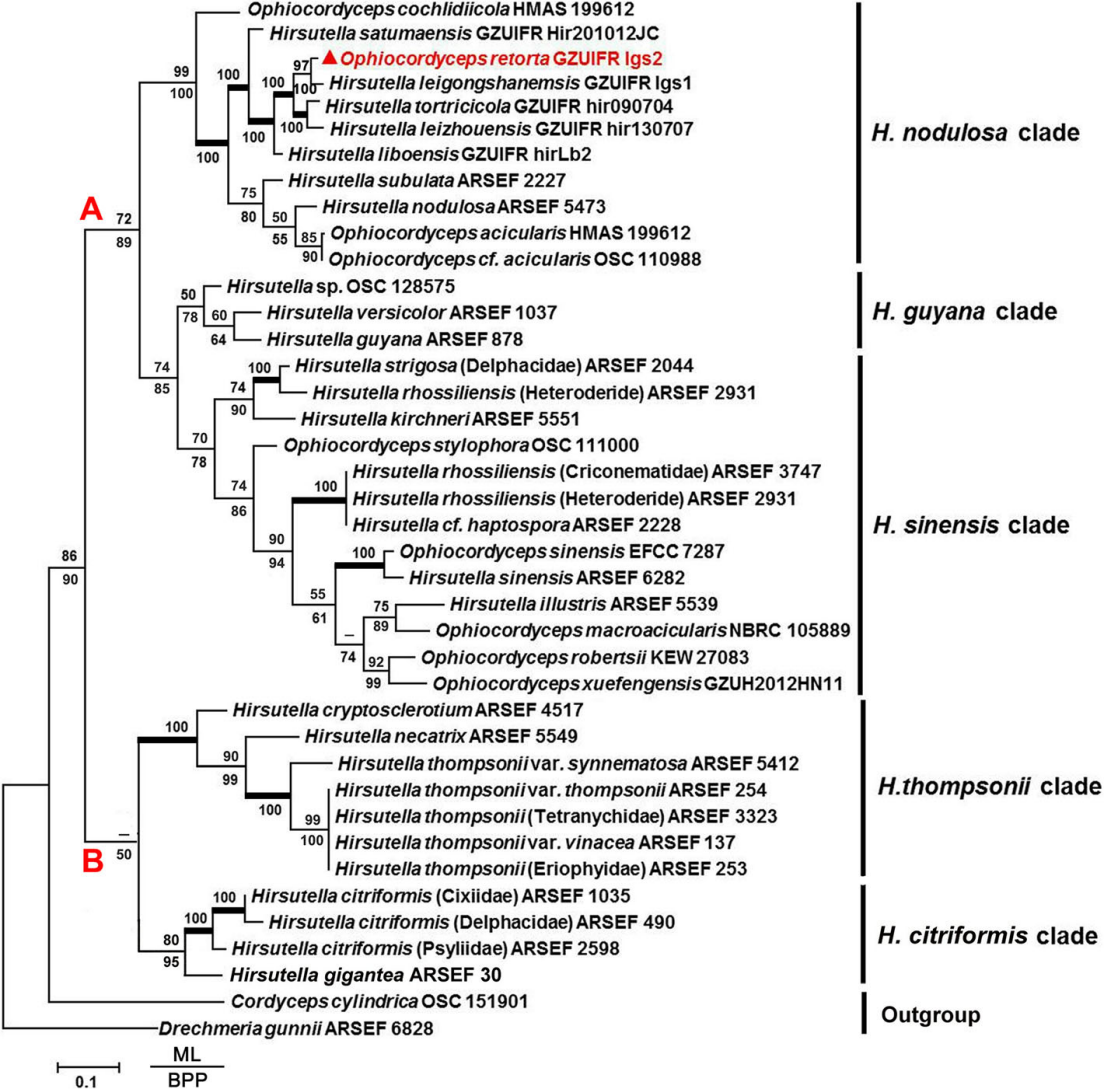
Phylogenetic tree of *Hirsutella* (asexual morphs) and *Ophiocordyceps* species combining TEF, ITS and 28S rDNA datasets obtained with Maximum likelihood method. Numbers below the branches are bootstrap percentage values based on 10,000 replicates. Bootstrap proportions of ≥50% are provided above corresponding nodes and in a thicker line, the branches in bold mean that ML/BPP both are 100%; the meaning of values above show as ML and the below are BPP near nodes; the BPP values<50% represent as “-”. The new species marked in red

A tree that was constructed using maximum likelihood and Bayesian posterior probabilities with *Drechmeria gunnii* and *Cordyceps cylindrica* as outgroups (Fig. 1) could be broadly divided into five taxa, which correspond to those in our previous studies [11, 21, 30]. The conidiogenous cell structures with asexual morph morphology were also demonstrated to be consistent with phylogenetic divisions. Our results indicated that the *Hirsutella* (asexual morphs) were restricted to two major groups, group A containing the clades *H. guyana*, *H. nodulosa* and *H. sinensis*, which occupied a long branch with strong support and displayed larger phialides, and group B with smaller phialide structures, including the clades *H. citriformis* and *H. thompsonii*.

The group A possess larger conidiogenous cells, verruculose apex, and orange segment-like conidiophores, including clade *H. guyana*, *H. nodulosa*, *H. sinensis* and the new species. The *H. nodulosa* clade contained all *Hirsutella* (asexual morph) species with a twisted neck and a bootstrap support of 99% ML/100% BPP. *O. acicularis*, which has been placed in the *H. sinensis* clade according to Simmons et al. [11], was shifted in clade *H. nodulosa* in this work, likely due to the inclusion of the ITS rDNA data. The *H. sinensis* clade included isolates originating from a variety of insect hosts, represented with *H. sinensis*, *H. strigosa* and *H. rhossiliensis*. The phylogenetic analysis placed the *H. guyana* clade as sister with *H. sinensis* clade with a bootstrap support of 74% ML/85% BPP.

The group B included *H. citriformis* and *H. thompsonii* clad with inflated, ovoid bases forming cymbiform or ellipsoid conidia. *H. citriformis* was the main represented species in *H. citriformis* clade, which is a widely distributed species infecting the Asian citrus psyllid *Diaphorina citri* [25]. The *H. citriformis* clade received bootstrap support values of 80% ML/95% BPP in the phylogenetic tree. The *H. thompsonii* clade, which was composed of five *H. thompsonii* species, *H. necatrix* and *H. cryptosclerotium*, and had a bootstrap support of 100% ML/100% BPP.

In addition, the new species *O. retorta* was clustered with *H. leigongshanensis* and acquired a strong bootstrap support (97% ML/100% BPP) in the *H. nodulosa* clade, which suggested that *O. retorta* is one member of the special group possessing helical twisting at the apex of phialides. However, despite the high bootstrap support, some differences in morphological characteristics were observed between *O. retorta* and *H. leigongshanensis*. *O. retorta* has two types of conidiogenous cells with large conidia (8.5–9.6 × 4.8–6 μm). More comparisons of morphological features with other related species was shown in Additional file 1: Table S2. Molecular phylogenetic analysis further confirmed the differences between the new species and other related species. Based on the molecular phylogeny together with morphological, biological and ecological characteristics, a distinct new *Ophiocordyceps* species is proposed.

### Morphological analysis

Phialide characteristics for the major *Hirsutella* asexual morphs clades reported in our phylogeny are summarized in Table 1.

**Table 1:** The main distinctive phialides characters of five clades

Clade	Phialides morphology	Representative species	Host
*H. thompsonii*	share short bottle-shaped or basally inflated, globose phialides, generally no more than 20 μm	*H. necatrix*, *H. gregis*, *H. cryptosclerotium*, *H. tydeicola*, *H. sphaerospora*	Acari, mite, Homoptera
*H. citriformis*	inflated bases and narrowly abruptly into a narrow long neck (20-40 μm)	*H. gigantea*, *H. fusiformis*, *H. radiata*, *H. guignardii*, *H. longicolla*, *H. saussurei*	Homoptera, Lepidoptera, Orthoptera, Diptera, Coleoptera
*H. nodulosa*	helical twisting at the apex of phialides and warts on base	*H. aphidis*, *H. brownorum*, *H. leizhouensis*, *H. liboensis*	Lepidoptera
*H. sinensis*	cylindric, slender or subulate base, gradually tapering to a warted neck, usually more than 30 μm	*H. sinensis*, *H. rhossiliensis*, *H. illustris*, *H. strigosa*	nematodes, mites, Hemiptera, Coleoptera, Lepidoptera
*H. guyana*	typical phialides, subulate with a basal cylindric portion, usually more than 30 μm	*H. guyana*, *H. versicolor*	Homoptera, Acari

#### *H. thompsonii* clade

*H. thompsonii* forms the core of a group of morphologically related species, including *H. cryptosclerotium*, *H. gregis*, *H. necatrix*, *H. sphaerospora* and *H. tydeicola*, pathogens of mites or nematodes, which display short bottle-shaped or basally inflated, globose phialides (generally no more than 20 μm), and subglobose conidia.

#### *H. citriformis* clade

The distinguished characteristics of this clade are inflated bases that narrow abruptly into a narrow long neck (20–40 μm), which mainly contains *H. gigantea*, *H. guignardii*, *H. fusiformis*, *H. longicolla*, *H. radiate*, *H. saussurei* and *Ophiocordyceps elongata*.

#### *H. nodulosa* clade

Clade *H. nodulosa* is a much special group of *Hirsutella* (asexual morphs) due to the distinctive helical twisting at the apex of phialides with warts on the base, which contains 11 members so far. However, *H. subulata* and several other *Ophiocordyceps* species without the twist structure are always clustered in this clade from phylogenetic topology analysis [11, 20]. One possible explanation is that the anamorphs of those *Ophiocordyceps* species might have a similar structure which has not been reported, or that lack of a twisted structure might be due to degeneration or loss during evolution.

#### *H. sinensis* clade

The *H. sinensis* clade have a complex range of hosts, including nematodes, mites and both hemi- (*Hemiptera*) and holometabolists (Coleoptera, Lepidoptera). The phialides of this group possess cylindric, slender or subulate bases, gradually tapering to a warted neck, usually more than 30 μm, but those of some species are exceptionally long and verruculose, even more than 100 μm, such as *H. strigosa* and *H. illustris*.

#### *H. guyana* clade

The *H. guyana* clade is represented by three *Hirsutella* isolates characterized in our study, which grouped with the *H. sinensis* clade and share similarly large conidiogenous cells and typical phialides, subulate with a basal cylindric portion.

### Dating and evolution of phialide structures in *Hirsutella* (asexual morphs)

We selected 40 main species with representative types of conidiogenous cells in *Hirsutella* (asexual morph) according to previous phylogenetic studies [11]. The data of nuclear genes individually provided limited information and could not perform a robust analysis. A Bayesian inference phylogenetic tree, which was constructed with a dataset consisting of 1703 bp from three genes and with a partitioning strategy, was established and yielded a clear hypothesis regarding the evolution of the different phialide structures in *Hirsutella* (asexual morphs) species (Fig. 1).

**Fig. 2:**
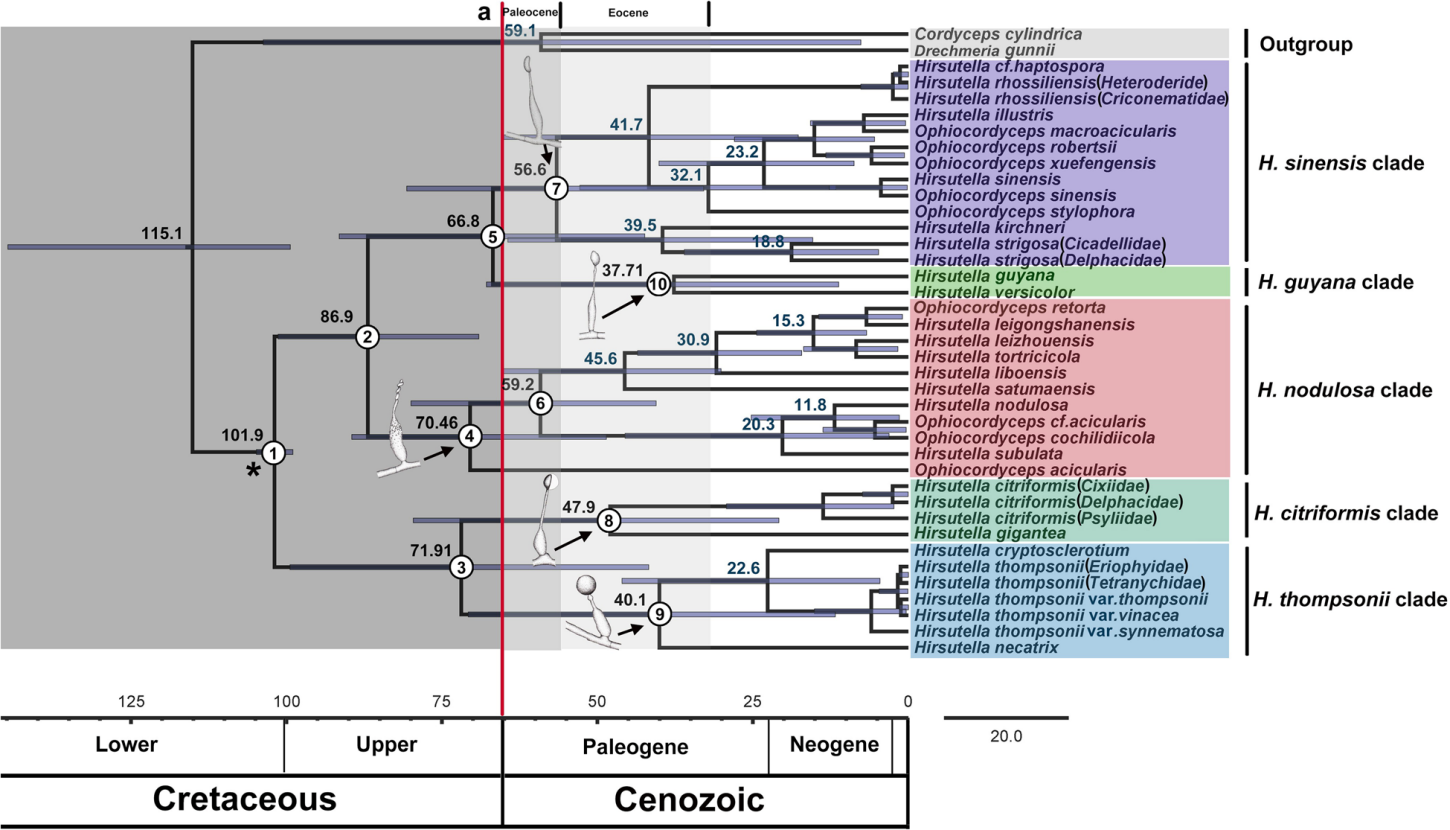
Divergence age estimates of major lineages of *Hirsutella* (asexual morphs). Chronogram was constructed based on the tree (Fig. 1) from maximum likelihood analyses. Geological times are provided below the chronogram and the dark gray rectangular box highlights the Cretaceous. Calibration (crown node of genus *Ophiocordyceps*) was based on *P. coccophagus*, a fungal parasite of a scale insect from the Early Cretaceous (Upper Albian) (99–105 Mya), marked with an asterisk “*”. Error bars are shown and each represents the 95% highest posterior density (HPD) for a node age. The red line “a” represents the mass extinction event of the Cretaceous–Tertiary (65.5 Mya)

In Fig. 2, the cluster of taxa with same phialide structure nearly coincided with the topology of the phylogenetic tree (Fig. 1), which provided an insight into the evolution of the internal phylogeny, origin, and evolutionary history of different types of phialide. Dating analyses support the supposition that the ancestor of the *Hirsutella* (asexual morphs) fungi was at least present in clade 1, that is, in the Early Cretaceous (Lower Albian) (101.9 Mya, 95% HPD: 99.0–104.9 Mya). The other clades distributed and diversified in the Upper Cretaceous (Fig. 2 and Table 2). Then, the new tree resolved the *Hirsutella* (asexual morphs) lineage into two major clades, 2 and 3, with topology corresponding to Fig. 1. The clade 2 included the *H. thompsonii* and *H. citriformis* clades, and clade 3 represented the *H. guyana*, *H. nodulosa* and *H. sinensis* clades. Since originated from a common ancestor, clade 2 and clade 3 were derived and evolved independently in parallel (Fig. 2). According to the clear divergence age estimates, clade 4 as well as clade 5 originated earlier as an independent group in the Early Maastrichtian (86.9 Mya, 95% HPD: 69.1–101.4). The age estimate of the crown node of clade 4 is at least 70.5 Mya (95% HPD: 48.3–90.0) in the Later Maastrichtian; and clade 5 diverged approximately 66.8 Mya (95% HPD: 42.2–91.8). In addition, clade 8 and clade 9 were originated later, at 71.9 Mya (95% HPD: 41.8–99.6 Mya), all sharing smaller phialide structures. The two clades centred at 47.9 Mya (95% HPD: 21.0–80.0) and 40.1 Mya (95% HPD: 11.9–71.6), respectively, around the Paleogene of Cenozoic (Fig. 2). Additionally, no major divergence of *Hirsutella* (asexual morphs) lineages was correlated with the Cretaceous–Tertiary extinction event (approximately 65.5 Mya). Therefore, a causal relationship between mass extinction events and the evolution of the phialides of the *Hirsutella* asexual morphs is not validated.

**Table 2:** Divergence time estimation of major clades of *Hirsutella* (asexual morph)

Node	Divergence time (Mya)	95% HPD range (Mya)
1	101.9	99.0–105.0
2	86.9	69.1–101.4
3	71.9	41.8–99.6
4	70.5	48.3–80.0
5	66 .8	42.2–91.8
6	59.2	40.6–60.1
7	56.6	33.2–80.9
8	47.9	21.0–80.0
9	40.1	11.9–71.6
10	37.7	11.0–68.2

### Taxonomy

**Fig. 3:**
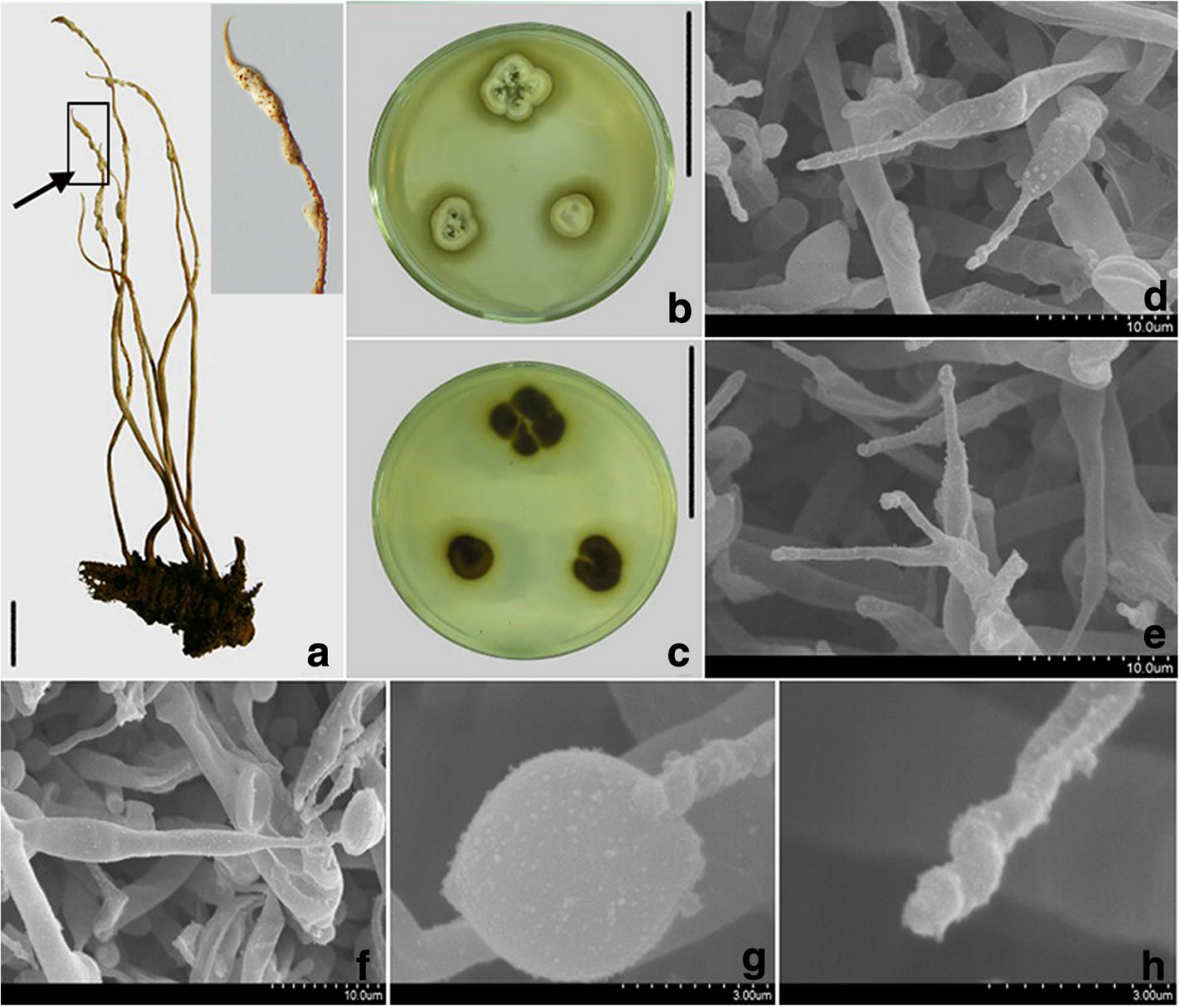
Morphological characteristics of *Ophiocordyceps retorta*. **a** Infection of the insect body specimen; **b**, **c** Colonial morphology on PDA agar medium for 20d, b showed the front of colony and c refer to the back of colony; **d**-**h**. SEM images showing conidiogenous cells and conidia structure; Bar: a = 1 cm, b, c = 5 cm; the rest of bar are shown in the figure

***Ophiocordyceps retorta*** X. Zou, J.J. Qu & Z.Q. Liang, *sp. nov*. Fig. 3.

MycoBank no.: MB 820078; GenBank no.: KY415594, KY415595, KY415597.

Type: China, Guizhou Province, Leigongshan Nature Reserve, hyphae emerging from a larval cadaver of a species of Cochlidiidae (Fig. 3a, 12 August, 2010, X. Zou. Holotype: GZUIFR-lgs2 and an isolated strain of its asexual stage GZUIFR-hir100812 were deposited at the Institute of Fungal Resources of Guizhou University (GZAC).

*Ophiocordyceps retorta* differs from related species mainly in its two types of phialides, verruculose, twisted or undulate neck.

Host was approximately 2.5 cm long × 0.9 cm wide. Synnemata were approximately 60–100 mm long × 0.3 mm wide, several, cylindrical, tapering at the end, arising from the thorax of the insect, sometimes simple-branched, brown, fawn yellow near the apex. The fungus hardly grows above 30 °C, while slowly spreads, with 15–23 mm dim. After 20 d on PDA agar under 20–22 °C (Fig. 3b&c). Colony was circular, dense and flat, centre of surface with light brown synnemata, pale yellow filiform aerial hyphae on colonies margins; light brown pigment secreting into the media making the back of the colonies show brown, 8–15 mm dim. Mycelium was hyaline, smooth verruculose and septate, with 2.1–3.5 μm wide. Conidiogenous cells with two types: A-phialides (Fig. 3d), 24–28.8 × 1 μm long, subulate with barely inflated base, 3–3.5 μm broad, tapering to a slender, verruculose and twisted neck, 9.6–10 μm long, 1 μm wide; and B-phialides (Fig. 3f), slender gourd-shaped or awl-shaped and tapered, width of base 3.5 × 1.5–2 μm, length 40.8–52.6 μm, width of neck 1.8–2.0 μm; both phialides types formed directly from the mycelium end, occasional polyphialidic. Conidia shaped like orange segments or oval, 8.5–9.6 × 4.8–6 μm, enveloped in hyaline mucus, thickness 1.8–2.7 μm (Fig. 3g).

Teleomorph: unknown.

Host: Larva of a species of Cochlidiidae, Lepidoptera.

Etymology: refers to the structure of the phialide neck, twisted (Lat. “retorta”).

Distribution: Leigongshan, Guizhou Province, China.

## Discussion

Previous studies with sequence data from additional genes demonstrated that *Hirsutella* (asexual morphs) are a monophyletic group with good statistical support [2, 29, 30], which was also supported by our phylogeny. Combining these results, it is apparent that phialides structure is both phylogenetically and taxonomically informative. Because the ITS sequence has a high resolution in the *Hirsutella* species distinction, it is necessary to add it into this phylogenetic analysis. However, the differences in topology of phylogenetic tree largely attributed to whether the ITS rDNA sequences are included or not. Further study is needed to confirm appropriate genes and polygenes of this genus. For example, our phylogenetic analysis removed *Ophiocordyceps acicularis* from the *H. sinensis* clade and placed it on the polytomy alongside *O.* cf. *acicularis* within the *H. nodulosa* clade. Simmons et al. describe that this species are not monophyletic, with representatives in both the *H. nodulosa* and *H. sinensis* clades [11]. However, we believed that this species has a close relationship with the twisted neck group. Further information is clearly required to resolve the relationships of these asexual morphs.

Sung et al. described an astonishing ascomycetous fossil, *Paleoophiocordyceps coccophagus*, in Burmese amber from 100 Mya, which is the oldest evidence of animal parasitism by fungi [29]. They firstly used the fossil and provided a minimum date for the age of the crown members of the Ophiocordycipitaceae, and then estimated that the Hypocreales as a whole were Jurassic in origin (150–200 Mya). Berbee et al. subsequently confirmed its accuracy and estimate an age of Ophiocordycipitaceae was 122 Mya (95% HPD: 109–138 Mya) [41]. Some additional examples of fungal pathogens in Dominican amber (30–45 Mya based on coccoliths) are synnemata produced by a *Hirsutella*-like fungus on a *Troctopsocopsis* bark louse [45]. The fossil was used for a crown calibration point for *Hirsutella* (asexual morphs) in Bayesian relaxed clock analyses and in age estimates of its crown group, dating it at least to the Early Cretaceous (101.9 Mya). Thus, we considered that the divergence times of *Hirsutella* asexual morphs are reliable.

The differentiation between phialides structures in major clades demonstrated a clear evolutionary path of *Hirsutella* species. The *Hirsutella* (asexual morphs) originated at the earliest, approximately 71.9 Mya in the Later Maastrichtian. The evolutionary trajectories of phialides followed two basic trends to host size with some degree of relationship. One trend, including lineages 8 and 9, has small phialides, irrespective of the presence of the host taxa used, usually being less than 25 μm and rarely with warts on the phialides. Conversely, another trend differs from the above in proliferation of phialide type, size range, and host diversity, which might increase the adhesive surface of conidia and sporulation efficiency. The nearly simultaneous origins that we observed for these two major lineages may be linked to the diversification of each clade into separate niches. In the current study, we did not find a direct relationship between *Hirsutella* (asexual morphs) and the Cretaceous-Tertiary extinction event [46]. This suggested that the diversity of phialides is more likely to be caused by long-term environmental adaptation and coevolution with insects, rather than dramatic extinction events. These origin and evolution results might be associated with the background of important biological and geological events in the Upper Cretaceous (approximately 60.0–100.5 Mya). Around the divergence times of *Hirsutella* (asexual morphs), the diversification of phialide events might accompanied with the abiotic stress of sea level and climate changes leading to high biotic stress conditions largely due to high nutrient levels, coevolving with increased plants and arthropods in late Cretaceous [47, 48].

The differences in the pattern of fungal evolution in regional communities may result from species differences in the relative importance of biotic interactions and coevolution [49–51]. With the angiosperm significant diversification between 115 and 90 Mya [52–56], a radiation of diverse monocot and eudicot lineages during this period began to modify the terrestrial ecosystems and initiate interactions with other organisms [57]. Cretaceous diversification of the pollinating insects, phytophagous insects and entomogenous fungi are consistent with the diversification of the angiosperms [29]. Similar to angiosperms, the exopterygotes and endopterygotes also radiated dramatically in the Cretaceous [58, 59]. Based on the functions of temporal and geographical distributions, all newly isolated *Hirsutella* species populations probably were forced into new coevolutionary pathways, with different types of conidiogenous cell structures evolving along new trajectories.

Coevolution between the fungus and its host insects might have originated a long time. It has been proposed that most groups of parasitic fungi probably evolved from saprophytic species developing on dead arthropods and plants [60, 61]. It has been demonstrated that plants are the potential hosts of *O. sinensis*, which seems to be able to jump from plants to insects and back onto a fungal host in accordance with the origins and evolution of entomopathogenicity [62, 63]. Such an evolutionary hypothesis might be used to explain the host specificity of *Hirsutella* (asexual morphs). It is reasonable to hypothesize that the host specificity of *Hirsutella* (asexual morphs) might relevant to the parallel evolutionary path and phialides diversification. Coevolution between the *Hirsutella* (asexual morphs) results in special physiological adaptations, which allow them to attack different insects or plants and lead to formation of a more suitable or advanced morphology of conidiogenous cells from the original structure. Such evolution in turn caused the special phialides structural differences that infect a group of related hosts. From an evolutionary point of view, the *H. thompsonii* and *H. citriformis* clades, sharing small hosts and simple phialides, originated earliest in *Hirsutella*, which might evolve an energy-efficient strategy to adapt niched of the host habitat. The subsequent evolution of lineages might result in producing more complex phialide structures, including the *H. nodulosa* and *H. sinensis* lineages. These results suggested that the diversity and large size of hosts possibly provided the basis for the formation of phialide complexity. For instance, the *H. nodulosa* clade, which originated in 70.5 Mya, might have increased the length of the neck to enhance the spread of spores by the dynamics of their ejection with twisted or undulated necks. It has been demonstrated that the subterminal part of phialide often display a helical or zigzag twisting in older cultures of fungi. Undulate and wavy tips are more pronounced in dead phialides, which reveals that the ecological functions of this structure are related to the spread of spores [64–66]. Therefore, we hypothesized that the twisted phialide neck might evolved independently under physiological adaptability to induce nutritional growth (access to nutrients, sunlight, water or space expansion better) or asexual reproduction (spore maturity and spread).

In *Hirsutella* (asexual morphs), another noted phenomenon is the polyphialidic characteristic, which is prevalent in various lineages, irrespective of the shape and size of phialides. This suggests that the evolution of the phialide structure is consistent with the progression from a simple to a more advanced form. It might also be an energy-efficient strategy to have one phialide with more than one locus of conidial development in a habitat, in which is a nutrition deficiency. In addition, a finer and more complex structure will always appear in the lineages with a large host, such as the helical neck, warted phialides, and whorled phialides, which are always present in the *H. nodulosa* clade or *H. sinensis* clade. More evidence, including additional fossil records, is needed to reveal the physiological function, ecological function, and phylogenetic position of this special phialide structure.

At present, the special group with a twisted neck of conidiogenous cells in *Hirsutella* (asexual morphs) species contains 11 members: *H. aphidis* Petch [67], *H. brownorum* Minter & B.L. Brady [68], *H. leizhouensis* H.M. Fang & S.M. Tan [69], *H. liboensis* Zou et al. [31], *H. nodulosa* Petch [24], *H. dendritica* Samson & H.C. Evans [70], *H. parasitica* Samson & H.C. Evans [70], *H. satumaensis* Aoki [71], *H. vermicola* M.C. Xiang & X.Z. Liu [72], *H. tortricicola* Zou et al. [73] and *H. leigongshanensis* Zou et al. [74]. In this study, we added a new member of the twisted phialide neck group, *Ophiocordyceps retorta* sp. nov. X. Zou, J.J. Qu & Z.Q. Liang. This species differs from related species mainly in its two types of phialides, verruculose, and twisted neck and its occurrence on larvae of Cochlidiidae (more morphological comparisons of the new species with other related species are shown in Additional file 1: Table S2).

## Conclusions

We have studied the pivotal internal phylogeny, morphological characteristics of phialide, origin and evolutionary history of the phialide structure in *Hirsutella* (asexual morphs). Based on phylogenetic and morphological analyses, five representative types of phialides, including the *H. citriformis* clade (representing by a dumpy ovoid base and a single slender neck), the *H. thompsonii* clade (with a cylindrical or round phialide that is small in shape, usually less than 25 μm), the *H. sinensis* clade (sharing a cylindrical base and an average phialide length of more than 40 μm), the *H. guyana* clade (typical phialides, subulate with a basal cylindric portion) and the *H. nodulosa* lineage (possessing phialides with apical helical twists) were firstly summarized that had a strong connection with the phylogenetic topology. The morphological characteristics and molecular genetic information could be productively combined for further generic classification studies, with great significance to the taxonomic study of *Hirsutella* (asexual morphs). The divergence of phialides structures in major clades demonstrated a clear evolutionary path of *Hirsutella* (asexual morphs), with a possible connection to host size that lead to two basic trends. One is elongation of the conidiogenous cells and increased complexity on auxiliary structures from the mycelium, and another is reducing the volume of phialides and spores, which might be linked to an energy-efficiency strategy. No major lineages was noted to be correlated with the nearby Cretaceous–Tertiary extinction event, which means that the diversity of phialides is more likely to be caused by long-term environmental adaptation and coevolution with insects rather than dramatic extinction events. These results suggested that the host specificity of *Hirsutella* asexual morphs might be relevant to the evolutionary pathways of phialides diversification.

## Additional file

Additional file 1: **Table S1.** GenBank accession numbers for sequences used in the phylogenetic analysis of *Hirsutella* (asexual morph). **Table S2.** Morphological comparison among *Ophiocordyceps retorta* and its related species. (DOC 86 kb)

## Abbreviations

AIC: The Akaike information criterion
BPP: Bayesian posterior probabilities
ESS: The measures of effective sample sizes
GZAC: The Institute of Fungal Resources of Guizhou University
HPD: The highest posterior density
ITS: ITS1–5.8S rDNA–ITS2 region, the first and the internal transcribed spacers
LM: Light microscope
LSU: The large subunits of the rDNA
MCMC: The Markov Chain Monte Carlo
ML: Maximum likelihood
Mya: Million years ago
PBS: Phosphate buffer solution
PCR: Polymerase chain reaction
PDA: The Potato Dextrose Agar
rDNA: Ribosomal DNA
SD: Standard deviation
SEM: Scanning Electron Microscope
TEF: The transcription elongation factor-1α
