# Supplementary material for: Estimated divergence times of Hirsutella (asexual morphs) in Ophiocordyceps provides insight into evolution of phialide structure
Source: BMC Evol Biol. 2018 Jul 13;18:111. doi: 10.1186/s12862-018-1223-0 (PMC6043951; doi:10.1186/s12862-018-1223-0)
Supplement: Supplementary file 1 — Table S1. GenBank accession numbers for sequences used in the phylogenetic analysis of Hirsutella (asexual morph). Table S2. Morphological comparison among Ophiocordyceps retorta and its related species. (DOC 86 kb) [file 12862_2018_1223_MOESM1_ESM.doc]

Table S1 GenBank accession numbers for sequences used in the phylogenetic analysis of *Hirsutella* (asexual morphs)

| Species | Isolate | *tef* (572 bp)* | ITS (560 bp)* | LSU (571 bp)* |
| --- | --- | --- | --- | --- |
|  |  | GTR+G^#^ | GTR+G^#^ | GTR+G^#^ |
| *Hirsutella leizhouensis* | GZUIFR-hir130707 | **KY415573** | **KY415580** | **KY415587** |
| *Hirsutella nodulosa* | ARSEF 5473 | KM652165 | KM652117 | KM652000 |
| *Hirsutella liboensis* | GZUIFR-hirLb2 | **KM652163** | **KM652115** | **KY415588** |
| *Hirsutella leigongshanensis* | GZUIFR-lgs1 | **KY415574** | **KY415581** | **KY415589** |
| *Hirsutella satumaensis* | ARSEF 996 | **KM652172** | **KM652125** | **KM652008** |
| *Hirsutella tortricicola* | GZUIFR-hir090704 | **KT368152** | **KY415583** | **KY415591** |
| ***Ophiocordyceps retorta*** | GZUIFR-hir100812 | **KY415594** | **KY415595** | **KY415597** |
| *Hirsutella lecaniicola* | ARSEF 8888 | KM652162 | KM652114 | KM651998 |
| *Hirsutella illustris* | ARSEF 5539 | KM652160 | KM652112 | KM651996 |
| *Hirsutella kirchneri* | ARSEF 5551 | KM652161 | KM652113 | KM651997 |
| *Hirsutella versicolor* | ARSEF 1037 | KJ524678 | KM652150 | KM652029 |
| *Hirsutella guyana* | ARSEF 878 | KM652158 | KM652111 | KM651994 |
| *Hirsutella haptospora* | ARSEF 2226 | KM651995 | KM652159 |  |
| *Hirsutella subulata* | ARSEF 2227 | KM652176 | KM652130 | KM652013 |
| *Hirsutella necatrix* | ARSEF 5549 | KM652164 | KM652116 | KM651999 |
| *Hirsutella gigantea* | ARSEF 30 | JX566980 | KJ524679 | JX566977 |
| *Hirsutella cf. haptospora* | ARSEF 2228 | KM652166 | KM652118 | KM652001 |
| *Hirsutella sinensis* | ARSEF 6282 | KM652173 | KM652126 | KM652010 |
| *Hirsutella strigosa* (Delphacidae) | ARSEF 2197 | KM652175 | KM652129 | KM652011 |
| *Hirsutella strigosa* (Cixiidae) | ARSEF 490 | KM652103 | KM651987 | KM652151 |
| *Hirsutella thompsonii* var*. thompsonii* | ARSEF 137 | KM652177 | KM652131 | KM652014 |
| *Hirsutella thompsonii* var*. vinacea* | ARSEF 254 | KM652194 | KM652149 | KM652028 |
| *Hirsutella thompsonii* (*Eriophyidae*) | ARSEF 253 | KM652179 | KM652133 | KM652016 |
| *Hirsutella thompsonii* (*Tetranychidae*) | ARSEF 3323 | KM652188 | KM652143 | KM652024 |
| *Hirsutella thompsonii* var*. synnematosa* | ARSEF 5412 | KM652193 | KM652148 | KM652027 |
| *Hirsutella citriformis* (*Delphacidae*) | ARSEF 490 | KM652151 | KM652103 | KM651987 |
| *Hirsutella citriformis* (*Cixiidae*) | ARSEF 1035 | KM652153 | KM652105 | KM651989 |
| *Hirsutella citriformis* (*Psyliidae*) | ARSEF 2598 | KM652155 | KM652107 | KM651991 |
| *Hirsutella rhossiliensis* (*Heteroderide*) | ARSEF 2931 | KM652168 | KM652121 | KM652004 |
| *Hirsutella rhossiliensis* (*Criconematidae*) | ARSEF 3747 | KM652170 | KM652123 | KM652006 |
| *Hirsutella cryptosclerotium* | ARSEF 4517 | KM652157 | KM652109 | KM651992 |
| *Ophiocordyceps robertsii* | KEW 27083 | KC561978 | EF468826 | EF468766 |
| *Ophiocordyceps stylophora* | OSC 111000 | JN049828 | JN941449 | DQ522337 |
| *Ophiocordyceps acicularis* | OSC 128580 | JN049820 | EF468805 | EF468744 |
| *Ophiocordyceps cf. acicularis* | OSC 110988 | GU723765 | DQ518757 | DQ522326 |
| *Ophiocordyceps xuefengensis* | GZUH2012HN11 | KC631800 | KX090288 | KC631791 |
| *Ophiocordyceps cochlidiicola* | HMAS 199612 | AB027377 | KJ878884 | KJ878965 |
| *Ophiocordyceps sinensis* | EFCC 7287 | HM140631 | KU239985 | EF468767 |
| *Ophiocordyceps macroacicularis* | NBRC 105889 | AB968402 | AB968418 | AB968576 |
| *Drechmeria gunnii* | ARSEF 6828 | HM140630 | HM140633 | AY489616 |
| *Cordyceps cylindrica* | OSC 151901 | KJ398817 | GU980044 | KJ878880 |

Note: * *tef*, transcriptional elongation factor 1-α; LSU, large subunits of the rDNA; ITS, the internal transcribed spacers (ITS1–5.8S rDNA–ITS2 region); the GenBank in bold were generated by this study.

^#^ Best nucleotide substitution model for each gene. G, gamma distribution; GTR, general time reversible.

ARSEF, USDA-ARS Collection of Entomopathogenic Fungal cultures, Ithaca, NY; OSC, Oregon State University Herbarium, Corvallis, OR; EFCC, Entomopathogenic Fungal Culture Collection, Chuncheon, Korea; NBRC, National Institute of Technology and Evaluation, Chiba, Japan; HMAS, Chinese Academy of Sciences, Beijing, China; KEW, mycology collection of Royal Botanical Garden, KEW, Surrey, UK; GZUIFR, Institute of Fungal Resources Collection, Guizhou University, China.

Table S2 Morphological comparison among *Ophiocordyceps retorta* and its related species

| Species | Phialides | Conidia | Host |
| --- | --- | --- | --- |
| *Hirsutella aphidis* petch | Awl | Cymbiform, 9×1.5*−*2.5μm | Aphid |
| *H. brownorum* Minter & B.L. Brady | Not polyphialidic | Lemon-shaped, 5*−*6×4*−*5μm | Mite |
| *H. dendritica* Samson & H.C. Evans | Wavy neck | Fusiform, non-mucoid, 6*−*8×2*−*3μm | Pupae |
| *H. leizhouensis* H.M. Fang & S.M.Tan | Rough-surfaced | Oviform, in mucoid sheath, 3.5*−*4.5×2*−*3μm | Phragmatoecia, castaneae |
| *H. liboensis* X. Zou, A.Y. Liu & Z.Q.Liang | Polyphialidic | Fusiform, in mucoid sheath, 6*−*10×1.5*−*4μm | Cossidae larva |
| *H. nodulosa* Petch | Rough-surfaced | In mucoid sheath, 3*−*5×3μm | Mite |
| *H. parasitica* Samson & H.C. Evans | Wavy neck | Cylindric, non-mucoid, 12*−*25×2.5*−*4μm | Aphid or scale insect |
| *H. satumaensis* Aoki | Rough-surfaced | Cymbiform or orange-segmented, 6*−*7×2.7*−*3μm | Bombyx mori |
| *H. vermicola* M.C. Xiang & X.Z. Liu | Singly or in opposite pairs | Orange-segmented, 7*−*8×1.5*−*3μm | Nematode |
| *H. tortricicola* X. Zou, Y.M. Zhou & Z.Q. Liang | Polyphialidic | Orange-segmented, 2.7*−*3.6×1.4*−*1.8μm | Tortricidae larva |
| *H. leigongshanensis* X. Zou, Y.L.Qing & Z.Q. Liang | Polyphialidic | Orange-segmented, 4.8*−*6×2.4*−*3.6μm | Grub larva  (*Coleoptera* sp.) |
| ***Ophiocordyceps retorta*** Zou *et al*. (**this work**) | A-phialides, subulate with barely inflated base; B-phialides, slender gourd-shaped and tapered. | Orange segments or oval, 8.5*−*9.6 × 4.8*−*6μm | Lepidoptera, Cochlidiidae |
